# Green Ferrate(VI) for Multiple Treatments of Fracturing Wastewater: Demulsification, Visbreaking, and Chemical Oxygen Demand Removal

**DOI:** 10.3390/ijms20081857

**Published:** 2019-04-15

**Authors:** Hongjing Han, Jinxin Li, Qin Ge, Yizhen Wang, Yanguang Chen, Baohui Wang

**Affiliations:** Institute of New Energy Chemistry and Environmental Science, College of Chemistry & Chemical Engineering, Northeast Petroleum University, Daqing 163318, China; hongjing_han@163.com (H.H.); jxli1012@126.com (J.L.); ky1994@126.com (Q.G.); aiyouweii99@163.com (Y.W.); ygchen79310@126.com (Y.C.)

**Keywords:** ferrate(VI), oxidation, demulsification, visbreaking, COD removal

## Abstract

Fracturing wastewater is often highly emulsified, viscous, and has a high chemical oxygen demand (COD), which makes it difficult to treat and recycle. Ferrate(VI) is a green oxidant that has a high redox potential and has been adopted for the efficient oxidation of fracturing wastewater to achieve triple effects: demulsification, visbreaking, and COD removal. Firstly, optimal conditions were identified to build a model for fast and efficient treatment. Secondly, wastewater treatment using ferrate oxidation was investigated via demulsification, visbreaking, and COD removal. Finally, a mechanism for ferrate oxidation was proposed for the three effects using Fourier-transform infrared (FT-IR) spectroscopy and scanning electron microscopy (SEM). The theoretical and experimental data demonstrated that the ferrate oxidation achieved the three desired effects. When ferrate was added, the demulsification efficiency increased from 56.2% to 91.8%, the total viscosity dropped from 1.45 cp to 1.10 cp, and the total removal rate of COD significantly increased to 74.2%. A mechanistic analysis showed that the strongly-oxidizing ferrate easily and efficiently oxidized the O/W interfacial film materials, viscous polymers, and compounds responsible for the COD, which was a promising result for the triple effects.

## 1. Introduction

Fracturing technology has been widely used to exploit low-permeability oil and gas reservoirs in recent years [1,2]. Using the principle of liquid transfer pressure [3], cracks occur when the speed of the fracturing fluid is larger than the injection velocity of formation absorption capacity [4]. In the area near the wellbore, the permeability of rock strata is improved so as to enhance oil and gas well production [5]. The fracturing fluid returns to the ground to produce the fracturing wastewater. The fracturing wastewater is characterized by complicated components, such as containing crude oil, organic additives, ceramsite sand, and other substances [6,7]. In addition to polycyclic aromatic hydrocarbons and benzene derivatives, other fluids are added to work synergistically in the untreated water that is produced [8]. These organics are stable and difficult to biodegrade, which creates large volumes of fracturing wastewater that have negative effects on the environment and human health [9]. Therefore, fracturing wastewater needs to meet a certain standard before the water is injected back into oil wells or otherwise discharged into the environment [10,11]. The current treatment methods include physical methods (gravity settlement [12], membrane filtration [13], ultrasonic separation [8], etc.), chemical methods (electrochemical oxidation [14], redox chemistry [15], neutralization [16], etc.), and biochemical processes (activated sludge [17], biofiltration [18], oxidation pond [19], etc.).

Ferrate is a strong inorganic oxidant and it a redox potential of 2.20 V under acidic conditions that has very broad applications in super batteries [20,21,22], wastewater treatment [23], green chemistry [24], etc. Ferrate(VI) is a highly-efficient wastewater treatment reagent that can be used as an effective oxidant, coagulant, demulsifier, disinfectant, and cathode material [25]. There has been an increasing amount of interest to activate Fe(VI) to enhance the oxidation of pharmaceuticals and pesticides [26]. In addition, ferrate(VI) can also rapidly oxidize inorganic and organic contaminants with the addition of one-electron and two-electron transfer reductants [27,28,29]. Some works proved that ferrate(VI) had better stability at the pH value of 10–11 [30,31] and some researchers have sought to improve ferrate(VI) activity at reduced or acidic pH [32,33]. Ferrate(VI) combined with ferric chloride was used to treat 12 different types of natural waters via the coagulation process, which confirmed that ferrate acts as a coagulant for water and wastewater treatment [34]. Ferrate(VI) was also used as an oxidant to eliminate organic pollutants [35] and as a medium to remove inorganic pollutants, such as metals and colloidal particles [36]. Ferrate technology offers a simple and efficient way to treat wastewater, and its use does not require changes to existing technological flowsheets, nor does it add large equipment, especially for the seasonal deterioration of water quality. Moreover, it does not produce carcinogenic organic pollutants and is harmless to the drinking water and is a new, efficient, and nonhazardous chemical reagent with a strong oxidability that shows great application prospects in wastewater treatment.

In this work, ferrate is first used to treat fracturing wastewater to explore a new treatment approach. The multiple actions including demulsification via oxidation, visbreaking, and COD removal are investigated and analyzed and a possible mechanism is proposed.

## 2. Results

### 2.1. Analysis of Fracturing Wastewater

The composition of fracturing wastewater is related to the components in the initial fracturing fluid, which contains varieties of chemical additives, such as gelatinizers and thickeners (1–2%, consisting of guar gum or polyacrylamide), a crosslinker (~0.2%, from borate), fluid loss control agents (0.2–0.5% originating from quartz sand or pottery clay), a gel breaker (~0.05%, consisting of persulfate), a chemical stabilizer and germicide (0.5–0.8%, formaldehyde and polyol etc.), a pH buffer (0.5–1%, sodium carbonate, citric, fumaric, etc.), and tiny amounts of surfactants. When the wastewater enters the ground, there are other organic and inorganic pollutants, including crude oil, high mineral ions, and bacteria [37].

For the initial fracturing wastewater, the COD value is 5190 mg/L, the suspended substance is 93 mg/L, the oil content is 1177 mg/L and viscosity is 1.45 cp, and the initial pH value is 5.6. The main components, gelatinizer, and thickener are about 1.0~2.0% of the total amount. Guar gum is the most abundant substance, and its content directly determines the viscosity and COD value of fracturing wastewater. Meanwhile, guar gum easily forms an emulsion with oil in the solution, which may make demulsification difficult. Therefore, the main pollutant is guar gum, and a reasonable emulsion model is the key to efficiently breaking the emulsion. The influences of pH, temperature, concentration of potassium ferrate, and reaction time are discussed in detail. It is necessary to investigate guar gum within the domain of demulsification to construct a model that can be used to quickly and efficiently treat fracturing wastewater.

### 2.2. Demulsification Efficiency by Potassium Ferrate Oxidation

**Figure 1:**
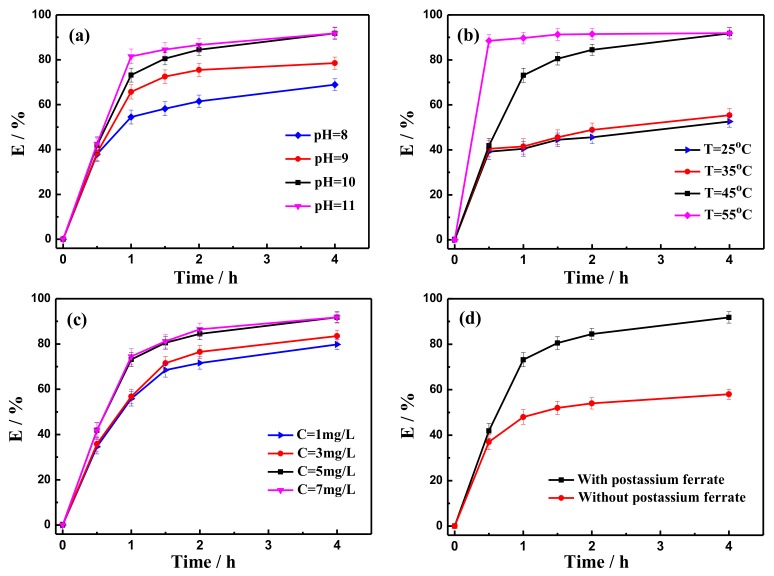
Demulsification efficiency of potassium ferrate under different conditions of (**a**) pH value, (**b**) temperature, (**c**), the addition of potassium ferrate, and (**d**) presence and absence of potassium ferrate.

The demulsification experiments using potassium ferrate under different conditions are illustrated in Figure 1. Figure 1a shows the demulsification efficiency with potassium ferrate at different pH values. For 4 h, as the pH value gradually increased, the demulsification efficiency increased from 42.5% at a pH of 8% to 91.8% at a pH of 10 and then remained almost unchanged at a pH of 11. This data shows that 10 was the optimal pH value. Figure 1b shows an obvious enhancement in the demulsification efficiency when the temperature was increased, reaching 91.8% at 45 °C and 55 °C for 4 h. This increase in efficiency occurred because the higher temperature increased the rate of droplet collision, which allowed the interfacial film to be broken more easily. Moreover, the reduction of solution viscosity is in favor of the settlement of the oil droplets. In the consideration of energy consumption, the optimal temperature was 45 °C. As displayed in Figure 1c, the demulsification efficiency increased rapidly and achieved a maximum (91.8%) when the potassium ferrate concentration was 5 mg/L, and then remained unchanged. Figure 1d shows that demulsification occurred when potassium ferrate was added, and the demulsification efficiency reached 91.8%. In contrast, the efficiency was just 56.2% in the absence of potassium ferrate. It resulted from the partial oxidation of organic compounds in the fracturing wastewater by potassium ferrate. In addition, the content of the suspended substance was also declined from 93 mg/L to 47 mg/L (Table 1). According to the experimental and economic benefits results, the conditions of 45 °C, a pH of 10, and a potassium ferrate concentration of 5 mg/L offered not only excellent demulsification performance but were also cost-effective.

**Figure 2:**
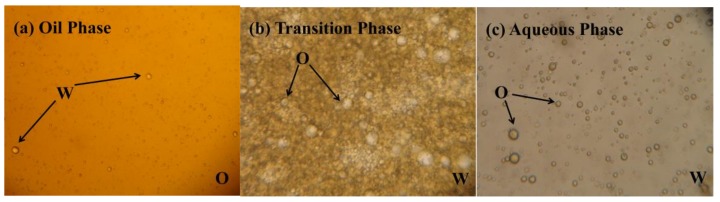
Morphological images of oil-water emulsification.

Figure 2 shows the morphological images of the oil-water (O/W) emulsion fluid at 4 h, and Figure 2a shows that the oil phase contained many small water drops, which formed the O/W emulsion system. In Figure 2b, the continuous phase was characterized by the dye method, which indicated that an O/W emulsion system was formed for the transition phase. Therefore, the smaller droplets should be oil. In Figure 2c, the aqueous phase contained many oil beads of different sizes, which formed an O/W emulsion system. After oxidation, the demulsification occurred, and the fracturing wastewater showed obvious stratification. Due to the separation of oil from the fracturing wastewater, the COD of the fracturing wastewater after demulsification was approximately 2476 mg/L, and the viscosity of the fracturing wastewater after demulsification was approximately 1.38 cp.

### 2.3. Visbreaking by Potassium Ferrate Oxidation after Demulsification

**Figure 3:**
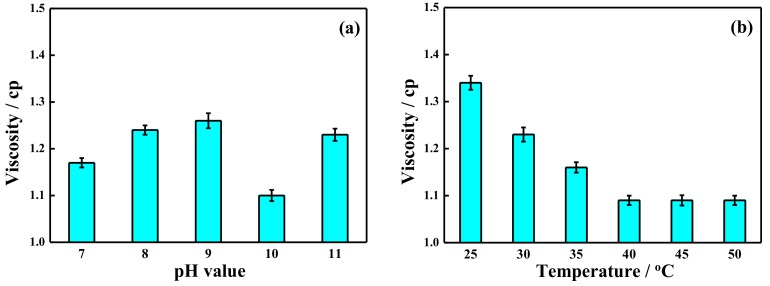

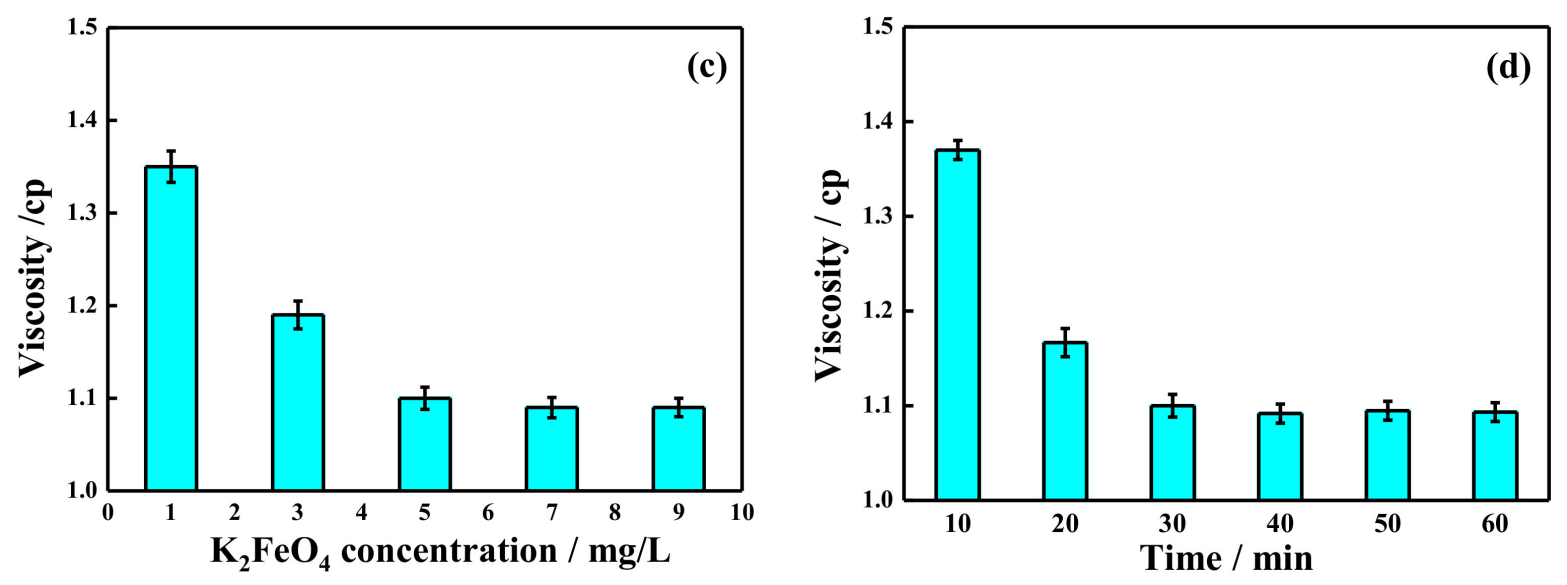
Visbreaking by potassium ferrate oxidation under different conditions of (**a**) pH value, (**b**) temperature, (**c**) the addition of potassium ferrate, and (**d**) reaction time.

Visbreaking by the oxidation of potassium ferrate is shown in Figure 3. Figure 3a displays the effects on visbreaking at different pH values when other experimental conditions were maintained at 40 °C, 5 mg/L potassium ferrate, and 30 min. During this experiment, the viscosity first decreased and then increased when pH increased from 7 to 10, and the lowest solution viscosity (1.10 cp) was recorded at a pH of 10. This trend can be explained by noting that the fracturing wastewater contains many different macromolecular and aromatic compounds, and a high redox potential is needed to degrade the chemical bonds in these organic molecules. The reduction potential and stability of potassium ferrate is the highest at a pH of 10, and it has enough time to react with the fracturing wastewater. However, at a pH of 11, the ability of potassium ferrate to degrade oxygen-containing cyclic compound became weak. The reason was that the amount of Fe(OH)_3_ colloid increased with pH value, which resulted in the reduction of the reaction between potassium ferrate and oxygen-containing cyclic compound, such as the rupture fragments of galactose and mannose. In Figure 3b, the effect of temperature was investigated under the conditions of a pH of 10, a 5 mg/L potassium ferrate concentration, and a 30 min reaction time. As the temperature increased, the viscosity gradually declined, and the viscosity reached a maximum value of 1.10 cp at 40 °C. The increased temperature caused the kinetic energy to increase, and the contacts between the potassium ferrate and fracturing wastewater were increased. The organics were quickly oxidized to rupture fragments or inorganics, which reduced the viscosity of the solution. As can be seen in Figure 3c, the effect of potassium ferrate concentration on visbreaking was examined under the conditions of a pH of 10, 40 °C, and 30 min. The results show that the viscosity decreased gradually as the concentration of potassium ferrate increased and then remained constant at concentrations higher than 5 mg/L. The strong oxidation of potassium ferrate and the flocculation effect of the decomposition product Fe^3+^ reduced the viscosity [38]. Figure 3d illustrates the effect of reaction time on visbreaking. When the time was extended to 30 min, the reduction of viscosity reached its minimum. Thus, the appropriate conditions were a reaction temperature of 40 °C, a pH of 10, a potassium ferrate concentration of 5 mg/L, and a reaction time of 30 min.

### 2.4. COD Removal Rate by Potassium Ferrate Oxidation after Demulsification

**Figure 4:**
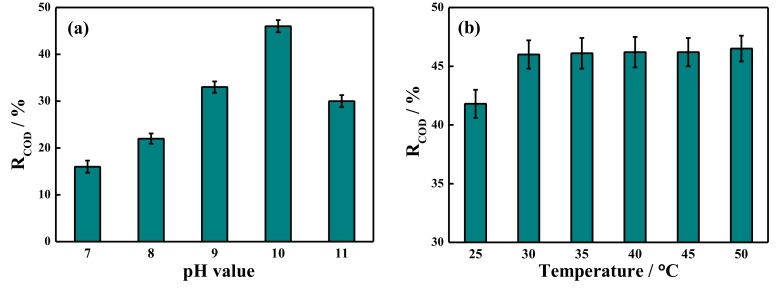

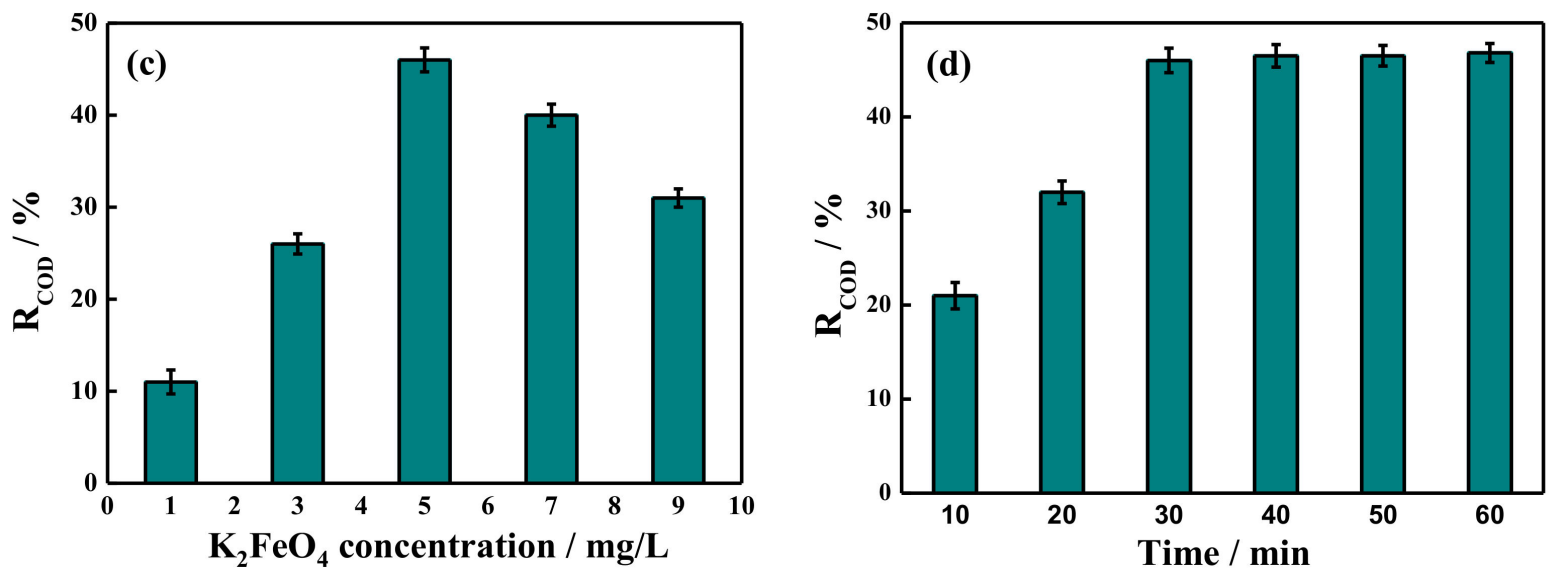
COD removal rate by potassium ferrate oxidation under different conditions of (**a**) pH value, (**b**) temperature, (**c**) the addition of potassium ferrate, and (**d**) reaction time.

The COD removal rate by potassium ferrate oxidation under different conditions is shown in Figure 4. As described in Figure 4a, the influence of pH on COD removal was performed at 40 °C, with 5 mg/L potassium ferrate, and a 30 min reaction time. The pH increased from 7 to 11, and the COD removal rate increased first and then decreased, reaching a maximum of 46% at a pH of 10. The effect of temperature was investigated, and the data is shown in Figure 4b. When the temperature increased, the removal rate of COD increased and then remained constant. Figure 4c shows the effect of the potassium ferrate concentration on COD removal at a pH of 10, 40 °C, and a 30 min reaction time. With the increase in concentration, the COD removal rate increased first and then decreased, and it reached a maximum COD removal rate of 46% using 5 mg/L potassium ferrate. Potassium ferrate was decomposed into Fe^3+^, which had the function of flocculation [38]. The flocculation increased with potassium ferrate and would slow down the reaction between potassium ferrate and oxygen-containing cyclic compound, which would result in the R_COD_ decrease when the concentration is higher than 5 mg/L. As shown in Figure 4d, the effect of reaction time was investigated at the same conditions. The COD removal rate increased and remained nearly constant at reaction times longer than 30 min, thus it was interpreted that the organic substances were oxidized and decomposed after 30 min. According to the visbreaking tests and the economic benefits, the best conditions for COD removal were determined to be a pH of 10, 40 °C, 5 mg/L potassium ferrate, and 30 min. Using these conditions, the COD value of the fracturing wastewater after treatment decreased from 2476 mg/L to 1337 mg/L, which conformed to the standards of produced water reinjection.

## 3. Discussion

### 3.1. Characterization of GG and Oxidized GG

**Figure 5:**
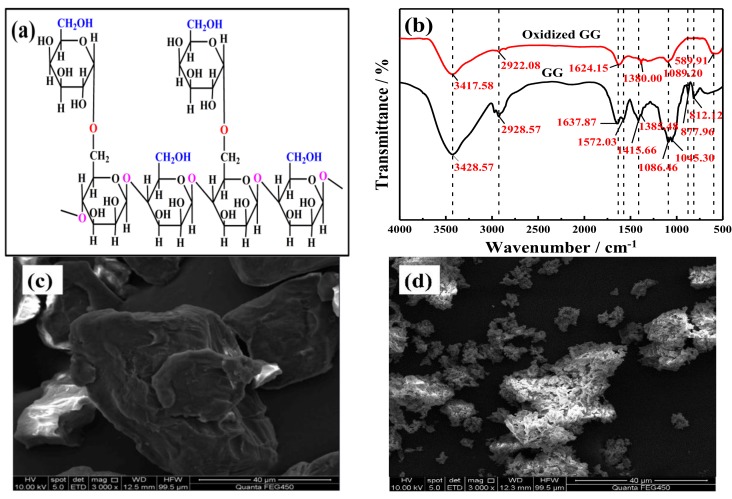
Characterization GG before and after oxidation with the (**a**) GG chemical structure, (**b**) FT-IR spectra (**c**,**d**), and SEM images of GG before and after oxidation.

Guar gum (GG) is the main organic substance in fracturing wastewater, and its chemical structure is shown in Figure 5a. The FT-IR spectra after oxidation are shown in Figure 5b, and IR wave numbers are summarized in Table 2. The peaks between 800 cm^−1^ and 1000 cm^−1^ were attributed to the highly-coupled C-C-O, C-OH, and C-O-C stretching vibrations of the polymer backbone. The absorbances of oxidized GG at 877.96 cm^−1^ and 812.12 cm^−1^ disappeared because the (1–4) and (1–6) linkages of galactose and mannose were disconnected. The reduction in the peak at 1400 cm^−1^ belonging to CH_2_ deformation and the weakened C-H stretching vibrations between 3000 cm^−1^ and 2800 cm^−1^ were due to the oxidation of CH_2_-OH. The region around 1640 cm^−1^ is due to the presence of ring stretching. In partially-oxidized GG, the absorption peaks around 1637.87 cm^−1^ were sharpened, which is associated with C-OH and indicated that the changes in the GG structure were not a result of the opening of the hexatomic ring but rather occurred because of the breakage of the main chain in the molecules. In Figure 5c,d, the unoxidized GG was an independent unit, and the surface was smooth. After oxidation, the main chains were oxidized and broken into small molecules, and single independent units were adsorbed and bridged together to form a single unit. The formerly smooth surface of oxidized GG was roughened and formed many jagged apertures.

Table 2 showed that the partial characteristic peaks disappeared after oxidation, and the presence of organic substances notably decreased in the solution. After oxidation, the peaks near 2928.57 cm^−1^ and 900 cm^−1^ disappeared, the hydroxyl stretch was weakened, and the flexural vibration peaks of ether bonds (C-O-C) and alcohols (C-O-H) at approximately 1045 cm^−1^ showed obvious decreases. The C-O-C and CH_2_-OH bonds were broken, which indicated that the galactose and mannose bonds were cleaved. The peaks near 500–700 cm^−1^ and 1637 cm^−1^ were still present, which showed that the six-membered rings were not broken or partially oxidized. Therefore, complex organic compounds were oxidized into small molecular fragments using potassium ferrate and these organic fragments were continually degraded. Afterwards, the chains were hydrolyzed, oxidized, and participated in other chemical reactions.

### 3.2. Possible Oxidation Mechanism for the Demulsification, Visbreaking and COD Removal

**Figure 6:**
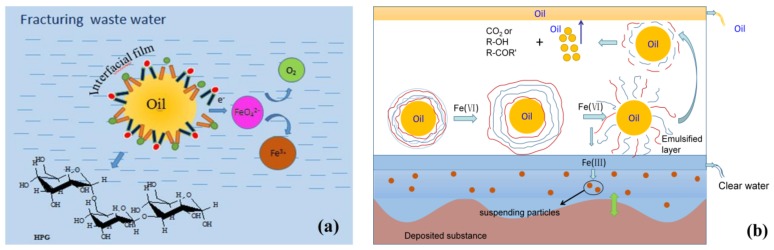
The possible mechanism of the oxidation process, showing (**a**) demulsification and (**b**) visbreaking.

Based on the experimental results, the possible oxidation demulsification mechanism of fracturing wastewater by ferrate is illustrated in Figure 6a, and is described as follows:

FeO_4_^2−^ + e → FeO_2_^−^ + O^2−^ + O• (1)

O^2−^ + H_2_O → 2OH^−^ (2)

FeO_2_^−^ + H_2_O → Fe (O)(OH) + OH^−^ (3)

2O• → O_2_↑ (4)

GG + H_2_O → HPG (5)

HPG → R-OH + e(6)

(HPG − Hydrolyzed GG)(7)

According to the emulsion model, oil droplets, which form an interfacial film in the fracturing wastewater, can be broken with ferrate. In alkaline solution, ferrate has a nice stability and can oxidize hydrolyzed GG in the interfacial film. FeO_4_^2−^ quickly reverts to ferric compounds [39,40]. Meanwhile, the hydroxypropyl guar gum (HPG) in the interfacial film and crude oil molecules lose electrons, which break the ether bonds and the hydroxyl bond of CH_2_-OH, which further oxidizes the organic compounds to produce aldehydes, ketones, carboxylic groups, carbon dioxide, and inorganics, and also releases oxygen.

According to the proposed demulsification mechanism model, the ferrate oxidized GG, which reduces the viscosity of the fracturing wastewater. A visbreaking model is proposed, which is illustrated in Figure 6b and shows that there are innumerable emulsion droplets in the fracturing wastewater. When potassium ferrate is added, the solution is demulsified, and the oil droplets enter the oil layer after demulsification. GG present in the interfacial film is gradually oxidized in the emulsifying layer, and changes from a long chain macromolecule compound to a short chain small molecular compound. Meanwhile, the reduced Fe^3+^ serves as an advanced flocculant to enhance treatment, and the suspended particles become deposits [41,42]. GG is continuously oxidized, and the viscosity of the solution continues to decrease, which causes the fracturing wastewater to display visbreaking and COD removal.

Oil is produced during the fracturing process and is mostly composed of crude oil. There are hydrocarbon chains and aromatics in the emulsion, and the possible gradual oxidation process of hydrocarbon chains is:

RCH_2_CH_3_ + FeO_4_^2^(OH) + H^+^ → RCH = CH_2_ → RCHCH_2_OH → RCH_2_CHO → RCH_2_COOH → RCH_3_ + CO_2_ (8)

RCH_3_ + FeO_4_^2^(OH) + H^+^ → RCH_2_OH → RCHO → RCOOH → CO_2_ (9)

Similarly, the possible mechanism for the gradual oxidation of large or branched aromatics is considered, and the final products are a variety of inorganic substances, such as N_2_, PO_4_^3−^, and so on. Thus, as the oil is degraded, the COD removal rate increases. Further research is needed to fully explore the details of the mechanism.

## 4. Materials and Methods

### 4.1. Chemicals and Materials

Potassium ferrate, sodium hydroxide, and potassium bichromate were received from the Harbin Chemical Reagent Co., China. Ammonium ferrous sulfate and sulphuric acid were purchased from Sinopharm Chemical Reagent Co., Shanghai, China. All chemicals were Analytical Reagent (AR) grade reagents, except sulphuric acid (chemically pure), and were used as received. Deionized water (D.I. H_2_O) was provided by a Hitech-Kflow water purification system (Hitech Co., Shanghai, China).

### 4.2. Sampling of the Fracturing Wastewater

The hydraulic fracturing wastewater was sampled from the Daqing oilfield in China. The samples were received by precleaned HDPE bottles with no head space, and stored at 4 °C prior to analysis. The fracturing wastewater had a light yellow-brown appearance accompanied by a strong pungent smell. Its viscosity was larger than that of water, and there was no obvious floating oil but there were clay particles.

### 4.3. Experimental Methods

#### 4.3.1. The Demulsification Test

After adjusting the pH value (initial pH value of 5.6) with NaOH solution, 2.5 mL potassium ferrate and 100 mL fracturing wastewater was thoroughly mixed in a 250 mL graduated cylinder. The mixture was shaken by a THZ-82 thermostatic water bath shaker (Wuhan Gray Mo Lai Detection Equipment Co., Wuhan, China) at 240 rpm for 2 h and left to stand for 24 h at 25 °C. The oil content of the aqueous phase was measured by an 8453 ultraviolet−visible (UV−vis) spectrometer (Hewlett-Packard Technology Co., Shanghai, China) monitoring the absorbance of 523 nm. The content of the suspended substance in the aqueous phase was measured by a JBXG-02 suspension solid determination instrument (Kunshan Guangxi Instrument Equipment Co. Ltd., Kunshan, China). The demulsification efficiency was calculated by the following equation.

E = (C_0_ − C)/C_0_ × 100% (10)

where E is the demulsification efficiency (%), C_0_ is the initial oil content (mg/L), and C is the oil content after the demulsifier solution was added.

#### 4.3.2. The Visbreaking Test

The fracturing wastewater after demulsification was stirred under 400 rpm with the addition of potassium ferrate, and the viscosity of the supernatant was measured by Ubbelohde viscometer (SYD-265D, Beijing Glass Instrument Factory, Beijing, China) every 3 min under 25 °C.

#### 4.3.3. The Measurement of COD

A given volume of the fracturing wastewater was measured by an LH-3C COD meter (Beijing Lianhua Yongxing Technology Development Co. Ltd., Beijing, China) after the treatment of potassium ferrate (2.5 mL) and LH-D (K_2_Cr_2_O_7_-HgSO_4_), LH-E (H_2_SO_4_-AgSO_4_) reagents were completely mixed in a glass tube, then digested for 10 min at 165 °C, cooled down to 25 °C and mixed with 2.5 mL D.I. H_2_O, and then the COD value. The removal rate of COD (R_COD_) was calculated by the following equation.

R_COD_ = (COD_after demulsification_ − COD_final_)/COD_after demulsification_ × 100% (11)

where COD_after demulsification_ was the COD value of the fracturing wastewater after demulsification in mg/L, COD_final_ was the COD value of the fracturing wastewater with the oxidation treatment.

### 4.4. Characterization

Scanning electron microscopy (Zeiss Sigma HV, Jena, Germany) was operated to observe the microstructure of guar gum (GG) and oxidized guar gum (oxidized GG).

Fourier-transform infrared (Vector-22, Bruker, Berlin, Germany) was recorded at room temperature in the range of 4000–400 cm^−1^ using KBr pellets.

Optical microscope (ECLIPSE80i, Nikon, Tokyo, Japan) was used for the morphology of oil-water emulsification.

## 5. Conclusions

A novel oxidation method employing ferrate was used to treat fracturing wastewater from oilfields. Guar gum was identified as the main substance that required treatment. The wastewater was characterized using FT-IR, SEM, and optical microscopy to obtain the following conclusions.

(1) Ferrate was highly efficient at demulsification. At 45 °C, a potassium ferrate concentration of 5 mg/L, a pH of 10, and a demulsification time of 4 h, the demulsification efficiency was 91.8%, and the COD decreased by 52.3% (from 5190 mg/L to 2476 mg/L), the viscosity decreased from 1.45 cp to 1.38 cp, and the content of the suspended substances declined from 93 mg/L to 47 mg/L.
(2) Ferrate oxidation was used for visbreaking and COD removal of fracturing wastewater after demulsification. The optimal conditions for treatment were determined to be 40 °C, a pH of 10, a potassium ferrate concentration of 5 mg/L, and a time of 30 min. The viscosity was reduced from 1.38 cp to 1.10 cp, the COD removal rate increased 46% (from 2476 mg/L to 1337 mg/L), and the quality of wastewater after treatment met the standard for produced water reinjection.
(3) According to the FT-IR and SEM analyses, a possible mechanism was introduced. Through the demulsification and the strong oxidation of ferrate, polymer chains in the oil-water interface films were broken, and the generated organic compounds were further degraded in the emulsified solution, which reduced the viscosity and COD value of the fracturing wastewater.

## Figures and Tables

**Table 1:** The suspended substance content of fracturing wastewater for different times.

Time (h)	0	0.5	1	1.5	2	4
Suspended substance (mg/L)	93	69.4	58.6	48.0	50.0	47.0

Reaction conditions: 45 °C, pH = 10, 5 mg/L potassium ferrate.

**Table 2:** Characteristic FT-IR wave numbers of GG and oxidized GG.

Characteristic Group	Wave Number (GG)	Wave Number (Oxidized GG)
O-H stretching vibration	3428.57	3417.58
C-H stretching of the CH_2_ group	2928.57	–
Ring stretching	1637.87	1624.15
C=O stretching vibration of COO^−^ group	1572.03/1415.66	–
Symmetrical deformations of the CH_2_ group	1385.48/1341.59	1380.00/1336.59
CH_2_OH primary alcoholic stretching mode	1086.46	1089.20
CH_2_ twisting vibration	1045.30	–
Galactose and mannose	877.96	–
The (1–4) and (1–6) linkages of galactose and mannose	960.26/812.12	–
Crystallinity of polymer	500~700	500~700
